# A type of magnetically induced self-assembled electrochemical aptamer nanobiosensor for ultrasensitive detection of hypoxia-inducible factor 1α protein

**DOI:** 10.3389/fchem.2026.1918181

**Published:** 2026-08-24

**Authors:** Xiangjun Zhou, Hezhong Ouyang, Liping Sui, Lei Sun, Xia Cao, Jingxuan Xu, Ruijiang Liu, Zhan-ao Wu

**Affiliations:** 1 Danyang Maternal and Child Health Hospital, Zhenjiang, China; 2 The People’s Hospital of Danyang, Affiliated Danyang Hospital of Nantong University, Zhenjiang, China; 3 School of Pharmacy, Jiangsu University, Zhenjiang, China; 4 Zhenjiang 359 Hospital, Zhenjiang, China

**Keywords:** aptamer, detection, electrochemistry, hypoxia-inducible factor 1α, nanobiosensor, α-Fe_2_O_3_/Fe_3_O_4_ magnetic heterogeneous nanorods

## Abstract

**Background:**

Hypoxia-inducible factor 1α (HIF-1α) protein has been identified as an aggressive malignant phenotype marker for numerous tumors; therefore, the detection of HIF-1α has become increasingly significant.

**Methods:**

In this work, we developed a type of magnetically induced self-assembled electrochemical aptamer nanobiosensor for detecting HIF-1α protein. This nanobiosensor utilized the magnetism of hydrothermal-calcination-synthesized α-ferric oxide/ferriferrous oxide (α-Fe_2_O_3_/Fe_3_O_4_, αFO/FFO) magnetic heterogeneous nanorods (MHNRs) to realize magnetically induced self-assembly; further, gold nanoparticles (AuNPs) were used to reinforce the electron transfer capacity and realize secure immobilization of the aptamer via Au–S bonds. Finally, we adopted cyclic voltammetry (CV), electrochemical impedance spectroscopy (EIS), and differential pulse voltammetry (DPV) for the electrochemical characterization, condition optimization, and performance analysis of the nanobiosensor, respectively.

**Results:**

The nanobiosensor revealed a favorable linear relation in the range of 0.1–1,000 ng/mL with a variance R^2^ of 0.995 and limit of detection of 0.112 ng/mL, excellent sensitivity, faithful reproducibility with a relative standard deviation (RSD) of 2.28%, reliable 13-d stability with an RSD of 1.71%, and accredited detection capacity for real samples with recoveries of 97.71%–107.47% and RSDs ≤ 3.47%.

**Conclusion:**

In this study, we developed a magnetically induced self-assembled electrochemical aptamer nanobiosensor for ultrasensitive detection of HIF-1α protein. The nanosensor delivers outstanding analytical performance with a facile assay strategy suitable for point-of-care testing, suggesting its promising prospects for clinical tests.

## 1 Introduction

Cancer remains the leading cause of mortality globally, with nearly 20 million new patients being confirmed every year and 9.7 million people dying from cancer annually (Zhou et al., 2026). Therefore, early and reliable diagnosis, precise delineation of the tumor and classification of its stage, and accurate prognostic treatment are pivotal for preventing cancer, optimizing the therapeutic schedule, and improving the survival rates of patients (Kumar and Jyoti, 2026). Hypoxia is a well-known and significant characteristic of the tumor environment owing to inadequate supply of physiological blood that results in the rapid growth of tumor cells. In response to this hypoxic state, the tumor cells promote the formation of factors that stimulate angiogenesis; these factors further induce the newly formed blood vessels to supply nutrients, resulting in further development of the tumor (Zielinska et al., 2024). Hypoxia-inducible factor 1α (HIF-1α) is one of the isoforms of the 92 kDa HIF protein and is one of factors that stimulate angiogenesis (Mukund et al., 2019); while HIF-1α promotes tumor growth, hypoxia due to the tumor also prompts the stabilization of HIF-1α (Swiatek et al., 2020). Consequently, HIF-1α is highly expressed in many types of cancers (Song et al., 2013), such as brain, stomach (Rohwer et al., 2010), colon, ovarian, kidney, hepatocellular (Zhuang et al., 2023), pancreatic, and bladder (Tam et al., 2020) cancers. Therefore, HIF-1α has been identified as an aggressive malignant phenotype marker for numerous tumors (Kappler et al., 2008; Vordermark and Brown, 2003).

Selected approaches have been developed for the detection of HIF-1α, such as those based on enzyme-linked immunosorbent assay (ELISA) (Zielinska et al., 2024), colorimetry (Wang et al., 2019), immunohistochemistry (Rosenberger et al., 2007), luminescence (Lu et al., 2016), rapid imaging (Ueda et al., 2010), and electrochemistry (Zhang et al., 2026). However, these techniques are often costly and/or laborious for routine application (Bonham et al., 2012; Zhang et al., 2012). With the development of nanoscience and nanotechnology, magnetic nanomaterials have offered novel promising methods and promoted newer technologies that have advantages such as lower toxicity, moderate magnetism, and superior stability (Alqarni, 2024; Materón et al., 2021). Among these, newer technologies involving magnetic self-assembled indicator-free electrochemical aptamer nanobiosensors have emerged owing to their need in recent times. Among magnetic nanomaterials, magnetic ferric-oxide-based nanomaterials have been widely applied in biomedicine owing to their heavy metal component and excellent electrical conductivity.

The frequently used ferric oxide nanomaterials mainly include those comprising α-Fe_2_O_3_ and Fe_3_O_4_; however, the low saturation magnetization (Ms) of α-Fe_2_O_3_ nanomaterials is disadvantageous for magnetic self-assembly, while the large Ms of Fe_3_O_4_ nanomaterials can result in birdnesting and disadvantages for surface modifications. Thus, a type of α-ferric oxide/ferriferrous oxide (α-Fe_2_O_3_/Fe_3_O_4_, αFO/FFO) magnetic heterogeneous nanomaterial (MHNM) was developed, whose moderate Ms effectively avoids the insufficiencies of α-Fe_2_O_3_ and Fe_3_O_4_ nanomaterials for electrochemical detection (Wang et al., 2025). Moreover, αFO/FFO magnetic heterogeneous nanorods (MHNRs) were developed to enhance the conductivity of such nanomaterials to aid electrochemical detection. Aptamers (Apts) are highly structured oligonucleotide chains (Xue et al., 2025) capable of specifically binding to, tracking, and inhibiting target molecules; these include various types, such as DNA aptamers, RNA aptamers, and peptide aptamers (Liu et al., 2013). Aptamer-based assays have garnered more attention in recent times and are being developed actively (Yadevan et al., 2025). Therefore, in this work, we fabricated αFO/FFO MHNRs via the novel hydrothermal method combined with the calcination-reduction process and modified the aptamers on their surfaces to construct a magnetically induced self-assembled electrochemical aptamer nanobiosensor for HIF-1α detection; finally, we investigated the detection capabilities of this nanobiosensor.

## 2 Experimental procedures

### 2.1 Chemicals and reagents

The aptamer of HIF-1α (sequence: 5′-SH-(CH_2_)_6_-TGTTGTTGTCTACGTGCT-3′) was purchased from Sangon Biotech Co., Ltd. (Shanghai, China), and the HIF-1α proteins were obtained from MedChemExpress Co. Ltd. (Shanghai, China); detailed information on the HIF-1α reference standard, including manufacturer, purity, storage and usage instructions, and batch number, are listed at the following link: https://www.medchemexpress.cn/recombinant-proteins/hif-1-alpha-protein-human-his.html. We procured FeCl_3_·6H_2_O, NH_4_H_2_PO_4_, glucose, Tris-base, HCl, NaOH, ethylene diamine tetraacetic acid (EDTA), K_3_Fe(CN)_6_, K_4_Fe(CN)_6_·3H_2_O, KCl, NaCl, HAuCl_4_·4H_2_O, C_6_H_5_Na_3_O_7_, and NaBH_4_ from Sinopharm Chemical Reagent Co., Ltd. Polyethyleneimine (PEI) was purchased from Macklin Biochemical Co., Ltd. (Shanghai, China). Tris-(2-carboxyethyl)-phosphine (TCEP) was obtained from Aladdin Reagent Co., Ltd. (Shanghai, China); 1× phosphate-buffered saline (PBS; pH 7.4) was obtained from Servicebio Technology Co. Ltd. (Wuhan, China). Bovine serum albumin (BSA) was procured from Saiguo Biotech Co., Ltd. (Guangzhou, China). Absolute ethanol was provided by Chengdu Chron Chemicals Co., Ltd. Finally, human serum samples were supplied by the Affiliated Danyang Hospital of Nantong University. No extra purifications were performed, and all analytical-grade reagents were utilized as obtained.

### 2.2 Preparation of αFO/FFO@Au magnetic nanocomposites

First, β-FeOOH nanorods (NRs) were fabricated by the hydrothermal method (Wang et al., 2024). In brief, 6 mg of NH_4_H_2_PO_4_ and 0.41 g of FeCl_3_·6H_2_O (molar ratio = 1:30) were added to 100 mL of ultrapure water (UPW) and magnetically stirred to form a homogeneous solution. Then, 70 mL of this solution was placed in a hydrothermal reactor and maintained for 12 h at 220 °C. After reaction completion, the hydrothermal reactor was naturally cooled to room temperature. The obtained suspension liquid was centrifuged, and the solids were washed in turn with UPW and absolute ethanol before being dried and ground to obtain the β-FeOOH NRs. Second, using the β-FeOOH NRs as the precursor, αFO/FFO MHNRs were fabricated by the calcination-reduction method. Briefly, the β-FeOOH NRs were mixed with glucose at a mass ratio of 1:16, and the mixture was calcined for 4 h at 500 °C in a furnace with programmed temperature control. After calcination, the furnace was cooled naturally and the resulting solids were ground to obtain the αFO/FFO MHNRs. Notably, the relevant synthesis conditions (molar/mass ratio, temperature, and time) were determined via a series of gradient optimization experiments. Both excessive and insufficient parameter values were observed to induce uneven dimensions, irregular NR shapes, and poor magnetic performance.

Third, 1.5 g of PEI was added to 150 mL of UPW and stirred until complete dissolution. Then, 50 mg of the αFO/FFO MHNRs were added and ultrasonicated for 30 min. The resulting suspension was placed in a flask and reacted for 90 min in a water bath at 90° C under magnetic stirring. After reaction completion, the resulting suspension was collected by centrifugation and the precipitate was rinsed twice using UPW; it was then dried in an oven and ground into a powder to obtain the αFO/FFO-PEI MNC. Finally, approximately 15 mg of αFO/FFO-PEI was added to 225 mL of UPW and ultrasonicated for 10 min in a low-temperature environment at the ice bath temperature. Then, 1 mL of HAuCl_4_ solution (20 mg/mL) was slowly dripped into the suspension and continuously ultrasonicated for 30 min in a low-temperature environment; moreover, 3 mL of sodium citrate solution (38 mM) was slowly dripped into the suspension while stirring and mixed for 1 min. Finally, 13 mL of a fresh sodium borohydride solution (0.075 wt%) was slowly dripped while stirring to reduce HAuCl_4_, and the stirring was continued for 15 min. After reaction completion, the resulting suspension was centrifuged and the precipitate was washed twice with UPW before being dried and ground to obtain the rod-like αFO/FFO@Au MNCs.

### 2.3 Construction of aptamer nanobiosensor and detection of HIF-1α protein

The aptamer was dissolved in Tris-EDTA (TE) buffer to obtain a 1 μM Apt solution; next, a TCEP solution in UPW (10 mM) was added to the Apt solution in the volume ratio of 1:100 (TCEP:Apt) to guarantee the presentation of sulfhydryl groups for immobilization via Au–S binding. Next, 15 μL of the αFO/FFO@Au suspension (20 mg/mL) was mixed with 15 μL of the Apt solution and incubated at 37° C for 3 h to form αFO/FFO@Au-Apt MNCs; this suspension was magnetically separated, and the solids of the αFO/FFO@Au-Apt MNCs were rinsed with PBS to remove the free Apt. The obtained αFO/FFO@Au-Apt MNCs were mixed with 30 μL of BSA (0.25%) solution in PBS and incubated at 25° C for 30 min to block the non-specific binding sites and obtain the αFO/FFO@Au-Apt/BSA aptamer nanobiosensor. Subsequently, the solid product was washed with PBS to remove excess BSA. Analogously, the obtained αFO/FFO@Au-Apt/BSA aptamer nanobiosensor was added to 30 μL of HIF-1α solution (10 ng/mL) and incubated for 30 min at 37° C to obtain αFO/FFO@Au-Apt/BSA-HIF-1α MNCs. All the obtained intermediate products were dispersed in 15 μL of UPW, and 9 μL of this suspension was dropped on a magnetic glassy carbon electrode (MGCE) polished with α-Al_2_O_3_ nanopowder. We then performed cyclic voltammetry (CV), electrochemical impedance spectroscopy (EIS), and differential pulse voltammetry (DPV) measurements (Bai et al., 2026) to examine the current signals using the CHI760 electrochemical workstation; here, the MGCE was used as the working electrode, Pt was used as the counter electrode, and Ag/AgCl was used as the reference electrode. The electrodes did not require a specific preservation environment and could be maintained at room temperature indoors. CV was carried out over a scanning voltage range of −0.1 to 0.7 V at a scan rate of 100 mV/s; EIS was performed in the frequency range of 0.1 Hz to 10 kHz at a signal amplitude of 5 mV; DPV was performed in the voltage range of −0.05 to 0.5 V. To enhance the detection sensitivity and accuracy, we optimized the key detection conditions involving the αFO/FFO@Au concentration, aptamer concentration, target incubation time, and target incubation temperature; we also investigated the analytical performances to reveal the selectivity, reproducibility, stability, and real sample detection capabilities. All DPV data were acquired from three independent parallel electrodes, and the error bars corresponded to the standard deviation (SD) of these replicate measurements (unless otherwise specified).

## 3 Results and discussion

### 3.1 Construction and detection principle of the aptamer nanobiosensor for HIF-1α

**FIGURE 1:**
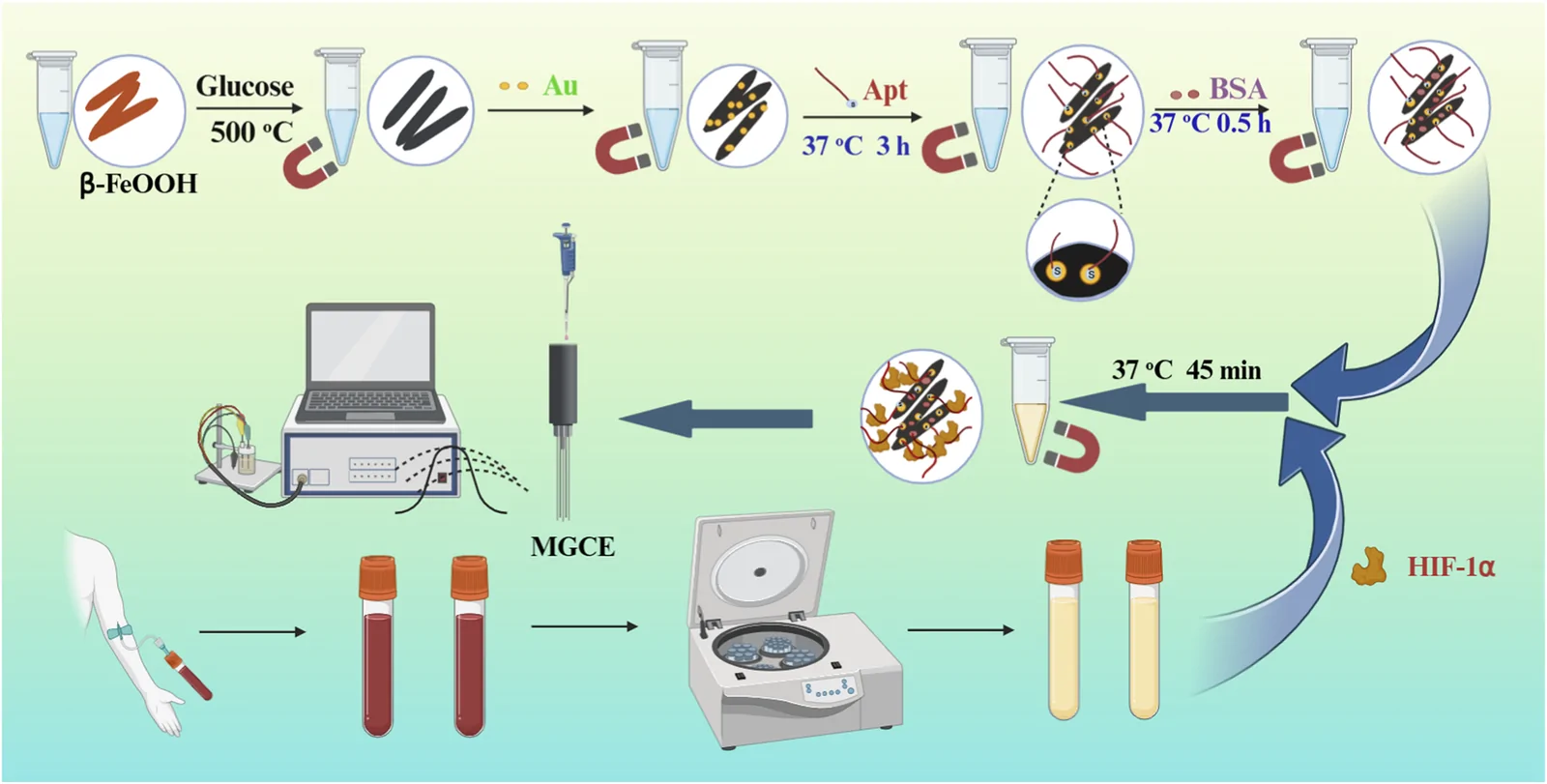
Schematic illustration of the electrochemical aptamer nanobiosensor for the detection of HIF-1α protein.

To realize the magnetically induced self-assembly property, αFO/FFO MHNMs were employed to construct the nanobiosensor; this effectively avoided any unfulfillable magnetically induced self-assembly owing to the low Ms of α-Fe_2_O_3_ and the birdnesting resulting from the large Ms of Fe_3_O_4_ that would hamper surface modification, thereby eliminating the troubles of labeled detection. For effective immobilization of the Apt, gold nanoparticles (AuNPs) were coated on the surfaces of the αFO/FFO MHNRs, and the Au–S bond was pivotal in securing the combination of αFO/FFO MHNRs and Apt containing sulfhydryl groups. To avoid the effects of non-specific sites on the detection of HIF-1α, BSA was employed to block the non-specific binding sites and produce the αFO/FFO@Au-Apt/BSA electrochemical aptamer nanobiosensor. Thus, as long as the HIF-1α molecules come into contact with the aptamer nanobiosensor, the HIF-1α proteins would be captured to form αFO/FFO@Au-Apt/BSA-HIF-1α MNCs. The changes in the electrochemical current signals for the αFO/FFO@Au-Apt/BSA aptamer nanobiosensor and αFO/FFO@Au-Apt/BSA-HIF-1α reflect the concentration of HIF-1α, which is the core principle. The schematic illustration for the construction of and detection using the aptamer nanobiosensor is shown in Figure 1.

### 3.2 Characteristics of the αFO/FFO MHNRs and αFO/FFO@Au MNCs

**FIGURE 2:**
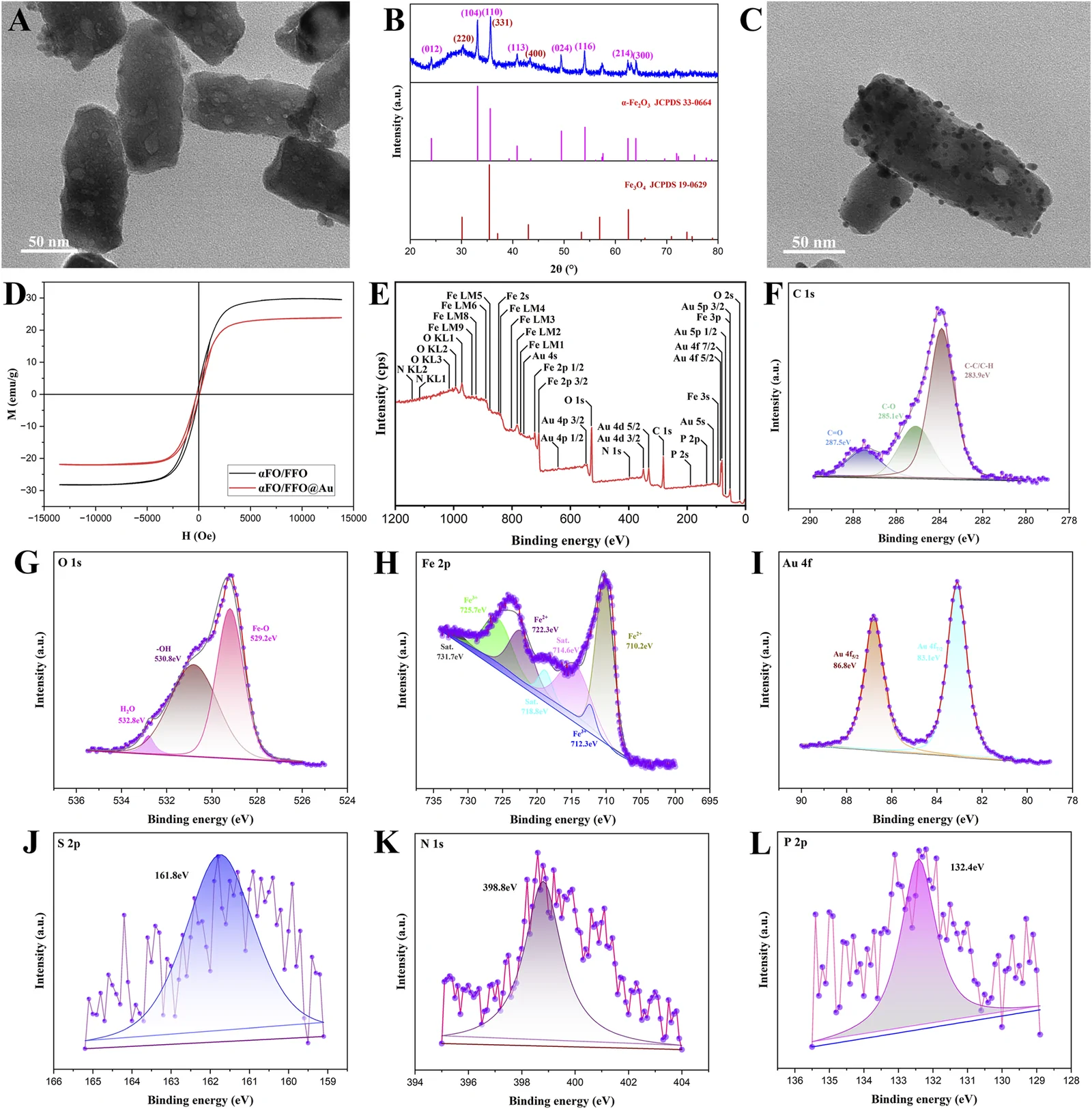
**(A)** Transmission electron microscopy (TEM) image and **(B)** X-ray diffraction pattern of the αFO/FFO magnetic heterogeneous nanorods (MHNRs); **(C)** TEM image of the αFO/FFO@Au magnetic nanocomposites (MNCs) and their **(D)** hysteresis loops; **(E–L)** X-ray photoelectron spectroscopy analyses of αFO/FFO@Au-Apt.

The morphology of the αFO/FFO MHNRs prepared via the hydrothermal process combined with the calcination-reduction process is displayed in Figure 2A via transmission emission microscopy (TEM); the sizes of the MHNRs were noted to be homogeneous, with average length and diameter values of roughly 135 and 55 nm, respectively. The X-ray diffraction (XRD) pattern of the αFO/FFO MHNRs is exhibited in Figure 2B, where the diffraction peaks at 24.1°, 33.1°, 35.7°, 40.9°, 49.5°, 54.0°, 62.4°, and 64.0° are, respectively, ascribed to the diffraction crystal planes of (012), (104), (110), (113), (024), (116), (214), and (300) for the α-Fe_2_O_3_ standard powder diffraction file (PDF) card (JCPDS 33-0664). However, the diffraction peaks at 30.4° and 43.4° do not correspond to any diffraction crystal planes of the α-Fe_2_O_3_ standard PDF card; moreover, the diffraction peaks at 33.1° and 35.7° are in the ratio of 0.975:1, which is inconsistent with the 1.429:1 ratio for the same diffraction angles of the α-Fe_2_O_3_ standard PDF card. The diffraction peaks at 30.4° and 43.4° correspond to the (220) and (400) diffraction crystal planes of the Fe_3_O_4_ standard PDF card (JCPDS 19-0629), suggesting the existence of both α-Fe_2_O_3_ and Fe_3_O_4_ in the final product. At the same time, the increase in the ratio of the diffraction peaks at 33.1° and 35.7° is attributable to the diffraction at 43.4° from Fe_3_O_4_, which proves the existence of Fe_3_O_4_ in the final product. Thus, the XRD pattern analysis results verify successful preparation of the αFO/FFO MHNRs.

The morphology of the αFO/FFO@Au MNCs is shown in the TEM image in Figure 2C, where the size of the αFO/FFO MHNRs remains almost unchanged while the AuNPs are uniformly immobilized on the surface and have an average diameter of approximately 6 nm. Figure 2D shows the hysteresis loops of the αFO/FFO MHNRs and αFO/FFO@Au MNCs, where the saturation magnetization values reach 29.8 and 23.9 emu/g, respectively; here, the saturation magnetization of the αFO/FFO@Au MNCs decreased by 5.9 emu/g compared to the MHNRs because of AuNP loading, which further proves the successfully preparation of the αFO/FFO@Au MNCs. X-ray photoelectron spectroscopy (XPS) characterizations (Figures 2E–L) were performed on αFO/FFO@Au-Apt to confirm the successful synthesis of αFO/FFO MHNRs, fabrication of αFO/FFO@Au MNCs, and aptamer immobilization. In Figure 2E, the atomic percentages of the constituent elements calculated from the XPS survey spectrum are as follows: Au (2.88 at.%), C (35.9 at.%), Fe (14.42 at.%), N (3.59 at%), O (38.51 at.%), P (2.85 at.%), and S (1.85 at.%). All XPS signals were calibrated using the adventitious carbon peak at 284.8 eV for charge correction.

The high-resolution Fe 2p spectrum (Figure 2H) exhibits two sets of spin-orbit doublets together with two characteristic satellite peaks for iron oxides. The doublet at 710.2 eV (Fe 2p_3/2_) and 722.3 eV (Fe 2p_1/2_) was assigned to the Fe^2+^ species within the Fe_3_O_4_ lattice, while the second doublet at 712.3 eV (Fe 2p_3/2_) and 725.7 eV (Fe 2p_1/2_) corresponded to Fe^3+^ originating from both the α-Fe_2_O_3_ and Fe_3_O_4_ phases. The two satellite peaks located at 718.8 and 731.7 eV verify the iron oxide nature of the nanocomposites, and the satellite peak at 731.7 eV arises from the undercoordinated hydroxyl-bound Fe^3+^ sites on the NR surfaces. The coexistence of Fe^2+^ and Fe^3+^ unambiguously confirms the intact heterostructure of the αFO/FFO MHNRs.

The O 1s spectrum (Figure 2G) fits into three distinct subpeaks; here, the dominant peak at 529.2 eV was assigned to the lattice Fe–O bonds, demonstrating the intact magnetic iron oxide substrate. The subpeak centered at 530.8 eV corresponds to the surface hydroxyl groups on the undercoordinated Fe sites. The peak intensity at 532.8 eV is significantly higher than that for the corresponding peak of the αFO/FFO@Au MNCs, indicating that it originates from physically adsorbed water molecules and P–O groups in the phosphate backbone of the thiol-terminated aptamer, indirectly verifying successful immobilization of the nucleic acid on the composite surface.

The Au 4f spectrum (Figure 2I) shows one set of spin-orbit doublets at 83.1 eV (Au 4f_7/2_) and 86.8 eV (Au 4f_5/2_), which was assigned to the bulk metallic Au^0^ inside the AuNPs. This Au^0^ component exhibited a negative shift of 0.9 eV relative to the standard bulk Au (84.0 eV) and originates from quantum confinement of the nanosized Au and electron transfer from the αFO/FFO MHNRs to Au. The formation of the Au–S covalent linkages was unambiguously verified through the S 2p spectrum (Figure 2J); here, the dominant S 2p_3/2_ peak centered at 161.8 eV is the characteristic binding energy of the thiolate sulfur chemically bonded to Au, along with the negligible signal of the unreacted free thiol (at approximately 163.5 eV). Meanwhile, the C 1s (Figure 2F), N 1s (Figure 2K), and P 2p (Figure 2L) spectra provide further evidence of successful aptamer immobilization. The C 1s spectrum can be deconvolved into three peaks from the DNA backbone: C–C/C–H (283.9 eV, aromatic and alkyl carbon), C–O (285.1 eV, ribose and phosphate), and C=O (287.5 eV, carbonyl groups in the nucleobases); the N 1s peak at 398.8 eV originates from the nitrogenous bases of the aptamer, and the P 2p peak centered at 132.4 eV corresponds to the phosphate diester groups of the nucleic acid. Collectively, these spectra unambiguously validate the heterogeneous architecture of the αFO/FFO MHNRs, AuNP modifications, and aptamer anchoring through the Au–S bond.

### 3.3 Properties of the detection system

**FIGURE 3:**
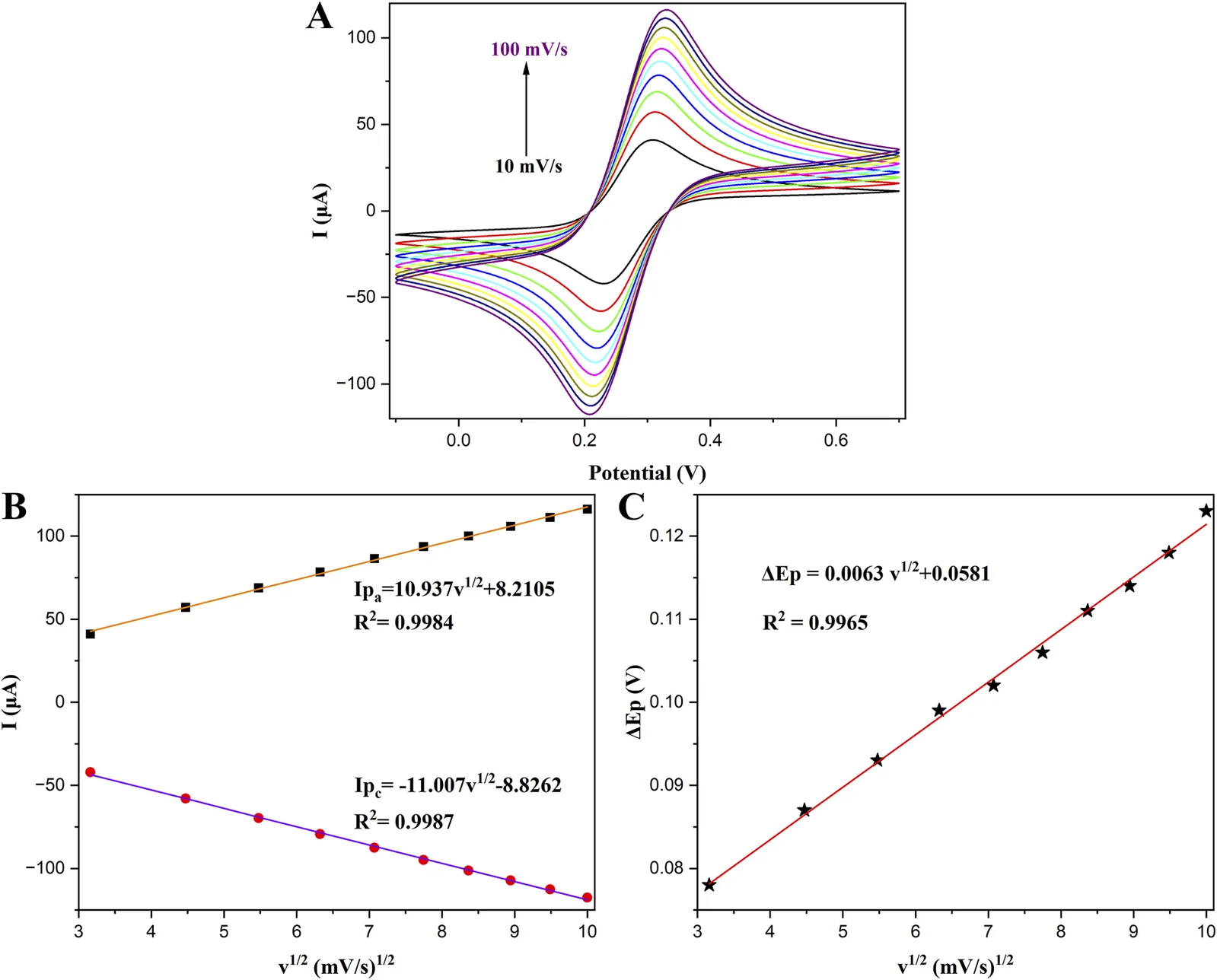
**(A)** Cyclic voltammetry (CV) curves, **(B)** anodic and cathodic peak currents, and **(C)** separation potential for the anodic to cathodic peaks of the bare magnetic glassy carbon electrode (MGCE) at various scan rates (10–100 mV/s).

In the nanobiosensor construction and detection process for HIF-1α determination, the MGCE with a diameter of 3 mm served as the working electrode while Ag/AgCl and Pt served as the reference and counter electrodes, respectively. The electrolyte containing 5 mM [Fe(CN)_6_]^3−/4−^ and 0.1 M KCl in UPW was used to detect HIF-1α on the CHI760E electrochemical workstation. Figure 3A shows the CV curves of the bare MGCE at various scan rates of 10–100 mV/s; as the scan rate increased from 10 mV/s to 100 mV/s, the redox peaks intensified progressively. The relationships of the peak anodic (Ip_a_) and cathodic (Ip_c_) currents vs. v^1/2^ are displayed in Figure 3B, and their simulated equations are given by Equations 1, 2.

$$Ip_{a}=10.937v^{1/2}+8.2105\;\left(R^{2}=0.9984\right),$$ (1)

$$Ip_{c}=-11.007v^{1/2}-8.8262\;\left(R^{2}=0.9987\right).$$ (2)

**FIGURE 4:**
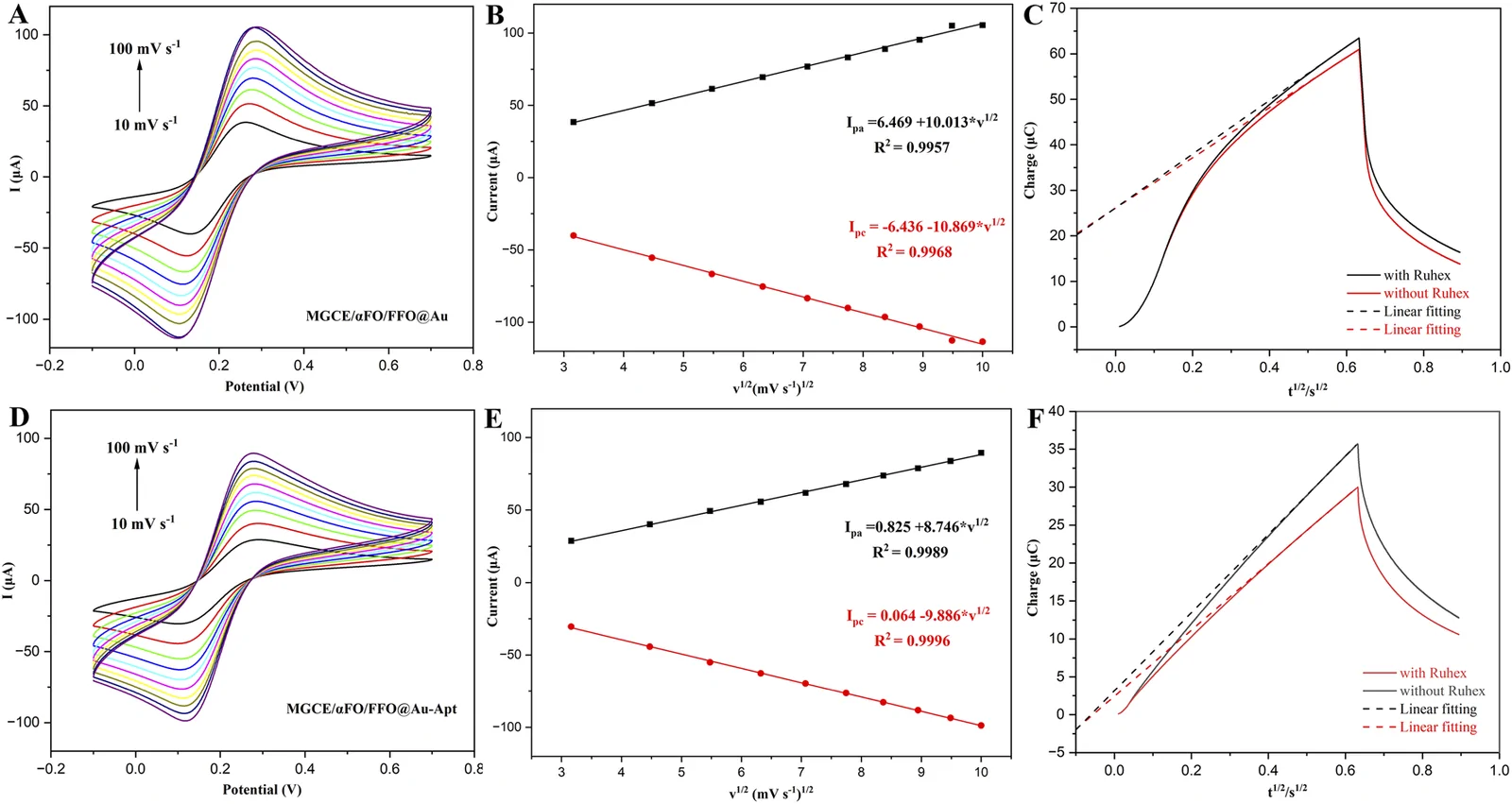
CV curves of **(A)** MGCE/αFO/FFO@Au and **(D)** MGCE/αFO/FFO@Au-Apt at various scan rates (10–100 mV/s); **(B, E)** linear variations of the faradaic process; **(C, F)** chronocoulometric curves in 0.1 M KCl solution containing 5.0 mM [Fe(CN)_6_]^3−/4−^ with and without 50 μM RuHex.

According to the Randles–Sevcik equation, *Ip = 2.69 × 10* ^*5*^ *n* ^*2/3*^ *AD* ^*1/2*^ *v* ^*1/2*^ *C* (where *n* is the electron transfer number, n = 1; *C* is the molar concentration of the redox active substance, 0.005 M; *A* is the effective cross-sectional area of the MGCE, 0.0707 cm^2^; *D* is the diffusion coefficient of the electrolyte) (Yue et al., 2025). The diffusion coefficient *D* was calculated as 0.013 cm^2^/s, which suggests the charge transfer ability of the electrolyte. The electrochemically active surface area of the MGCE/αFO/FFO@Au was calculated as 0.0647 cm^2^ (Figures 4A,B), while that of the MGCE/αFO/FFO@Au-Apt was calculated as 0.0565 cm^2^ (Figures 4D,E).

To understand the reversible or irreversible nature of the electrochemical reaction at the interface, the potential differences (ΔEp) corresponding to Ip_a_ and Ip_c_ were calculated at various scan rates. Figure 3C depicts ΔEp and its dependence on the square root of the scan rate, where all the ΔEp values are noted to be greater than 0.078 V, indicating a quasi-reversible electrolysis kinetics (Saidi et al., 2025). This reveals the good linear relationship of ΔEp vs. v^1/2^ with R^2^ = 0.9965, and ΔEp increases with increase in v, indicating a diffusion-mediated reaction kinetics for the electrochemical detection (Turunc et al., 2026). Therefore, electrochemical detection using [Fe(CN)_6_]^3−/4−^ and KCl as the electrolyte belongs to the diffusion-mediated quasi-reversible electrolysis process. The corresponding linear fitting equation is given in Equation 3.

$$\Delta Ep=0.0063v^{1/2}+0.0581\;\left(R^{2}=0.9965\right).$$ (3)

### 3.4 Feasibility of the electrochemical aptamer nanobiosensor for HIF-1α protein detection

**FIGURE 5:**
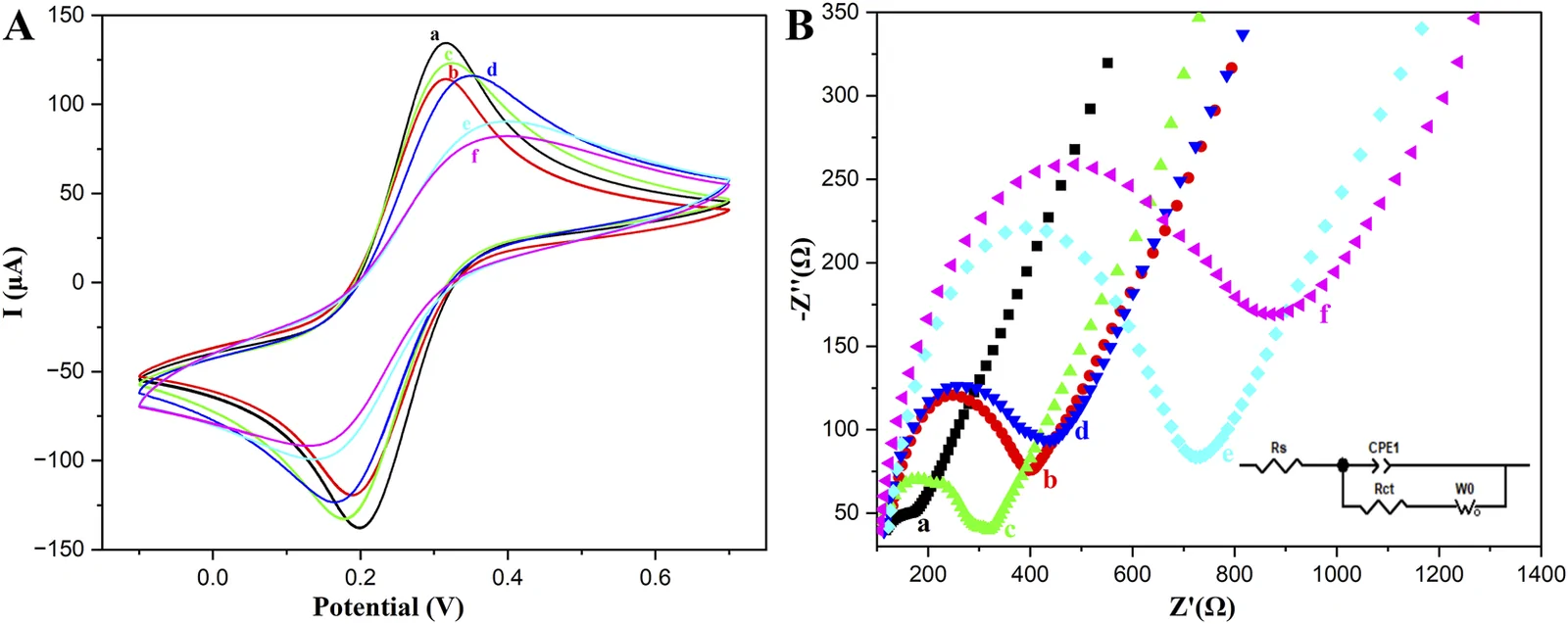
**(A)** CV curves and **(B)** Nyquist plots of the bare and different modified electrodes: **(a)** bare MGCE, **(b)** MGCE/αFO/FFO, **(c)** MGCE/αFO/FFO@Au, **(d)** MGCE/αFO/FFO@Au-Apt, **(e)** MGCE/αFO/FFO@Au-Apt/BSA, and **(f)** MGCE/αFO/FFO@Au-Apt/BSA-HIF-1α.

The CV curves and Nyquist plots are illustrated in Figure 5A, where the CV measurement for the bare MGCE (curve a) exhibits well-defined redox peaks. The CV curve for the MGCE dripped with the αFO/FFO MHNRs (MGCE/αFO/FFO) is shown in curve b; here, the peak current decreases compared to curve a for the bare MGCE, which could be due to the relatively low electrical conductivity of the αFO/FFO MHNRs, leading to diminished electron transfer and a reduced current response. In contrast, the CV measurement for MGCE/αFO/FFO@Au (curve c) exhibits a substantially enhanced peak current than that of MGCE/αFO/FFO; this improvement is consistent with the previously mentioned high conductivity of the AuNPs, which facilitate electron transfer and thereby amplify the current signal. A further decrease in the peak current was recorded for the MGCE/αFO/FFO@Au-Apt (curve d), indicating successful immobilization of Apt on the surface of αFO/FFO@Au. The peak current of MGCE/αFO/FFO@Au-Apt/BSA (curve e) was lower than that of MGCE/αFO/FFO@Au-Apt, which could be explained by the steric hindrance caused by BSA through the blockage of non-specific sites. Subsequently, the peak current of MGCE/αFO/FFO@Au-Apt/BSA-HIF-1α (curve f) decreased further, and the reason for this was the electron transfer capacity of HIF-1α. These changes form the basis for detecting HIF-1α and are the most important design principle of the electrochemical aptamer nanobiosensor.

The EIS detection data are illustrated in Figure 5B. The semicircular section in the Nyquist plot typically reflects the charge transfer resistance (R_ct_), with a larger semicircular radius reflecting a higher R_ct_ value; the simulated electrochemical parameters of the various modification steps are presented in Table 1. Figure 5B clearly shows that the R_ct_ of MGCE/αFO/FFO (186.9 Ω) is obviously larger than that of bare MGCE (99.5 Ω); however, the R_ct_ of MGCE/αFO/FFO@Au (153.1 Ω) is lower owing to AuNP loading. Thereafter, with the sequential loading of Apt, BSA, and HIF-1α, the R_ct_ values increase to 251.5 Ω, 285.6 Ω, and 327.4 Ω, respectively. These findings are in agreement with those from the CV analysis.

**TABLE 1:** Electrochemical parameters of different modification steps for HIF-1α detection.

Electrode	R_s_ (Ω)	R_ct_ (Ω)	W_o_ (σ)	C_d_ (μF)	τ (s)	Chi-squared	Sum of squared value
Bare MGCE	134.9	99.5	0.40790	0.83141	82.72	9.3433 × 10^−4^	0.10745
MGCE/αFO/FFO	91.34	186.9	0.44870	0.84709	158.32	7.7867 × 10^−4^	0.089547
MGCE/αFO/FFO@Au	147.5	153.1	0.43920	0.83991	128.59	1.0782 × 10^−4^	0.012399
MGCE/αFO/FFO@Au-Apt	130.3	251.5	0.40597	0.83273	209.43	3.5481 × 10^−4^	0.040803
MGCE/αFO/FFO@Au-Apt/BSA	131.6	285.6	0.39854	0.83183	237.57	4.8459 × 10^−4^	0.055728
MGCE/αFO/FFO@Au-Apt/BSA-HIF-1α	132.0	327.4	0.39201	0.82766	270.98	2.9578 × 10^−4^	0.034015

### 3.5 Chronocoulometry for aptamer quantification

To evaluate the immobilization efficiency and sensing potential of the biosensor, we performed chronocoulometry to quantify the surface-immobilized aptamers. Initially, the net adsorption charge (ΔQ) was obtained by subtracting the intercepts of the Q vs. t^1/2^ curves (Figures 4C,F); subsequently, the surface coverage of the electrode-bound aptamer (Γ_ss_) was quantified via Equation 4:

$$\Gamma_{\text{ss}}=\frac{\Delta QN_{A}}{nFA}\cdot \frac{z}{m},$$ (4)

where N_A_ is Avogadro’s number, n is the number of electrons (n = 1), F is Faraday’s constant, *A* is the electrochemically active surface area, z is the charge of the redox marker (equal to 3), and m is the number of nucleotides in the DNA (equal to 18). Ultimately, Γ_ss_ was calculated to be 1.30 × 10^13^ per cm^2^.

### 3.6 Optimization of the construction conditions for the aptamer nanobiosensor

**FIGURE 6:**
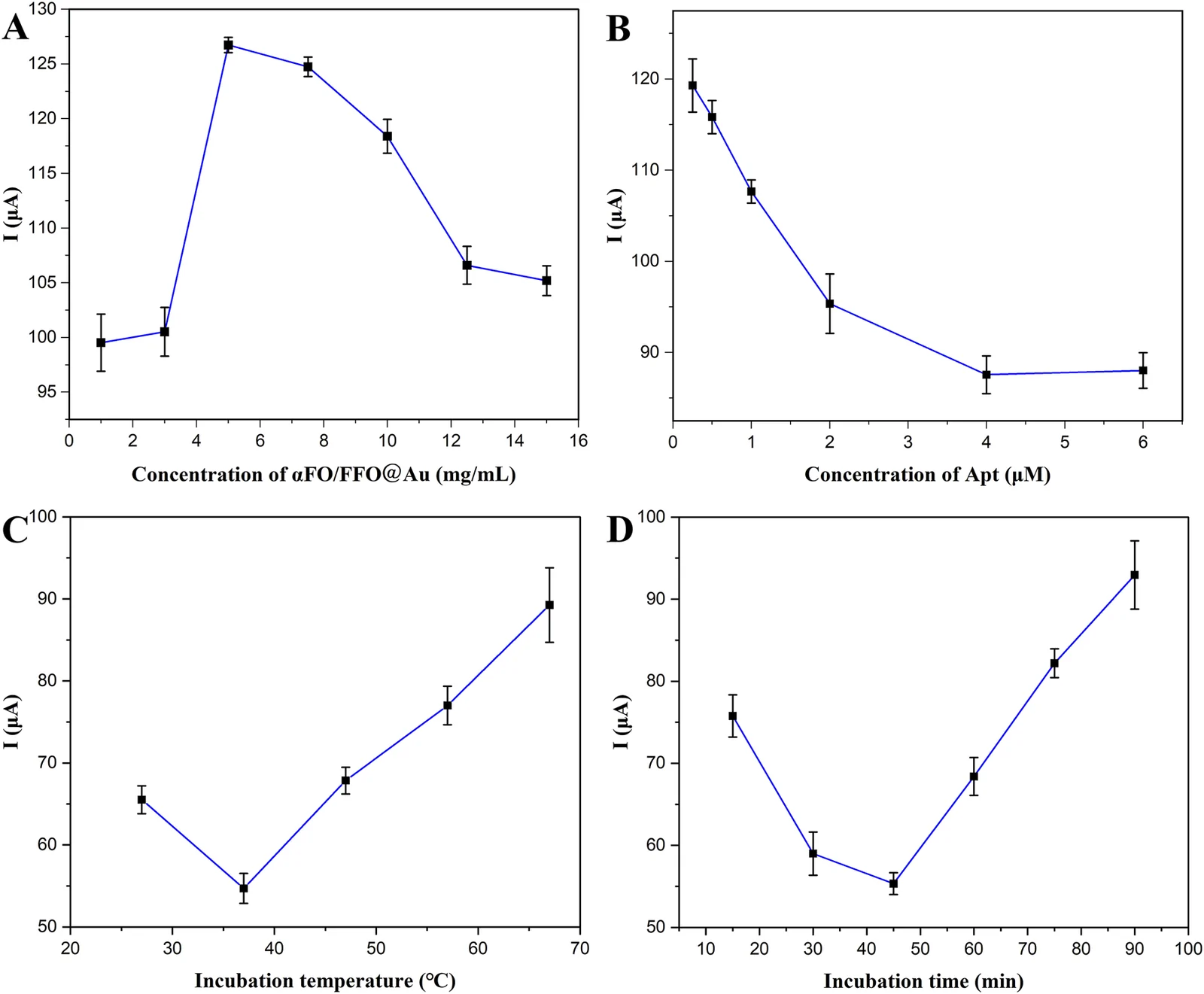
Optimal **(A)** concentration of αFO/FFO@Au for maximum conductivity, **(B)** target concentration of Apt, **(C)** incubation temperature of HIF-1α, and **(D)** incubation time of HIF-1α for preserving protein performance.

For more sensitive detection of HIF-1α, four pivotal parameters in the construction of the electrochemical aptamer sensor were optimized by DPV, namely, the concentration of αFO/FFO@Au, concentration of Apt, incubation temperature of HIF-1α, and incubation time of HIF-1α; these optimizations are summarized in Figure 6. The optimization result for the concentration of αFO/FFO@Au is shown in Figure 6A; here, the peak current initially increases and subsequently decreases with increase in concentration, reaching a maximum at the concentration of 5 mg/mL. The initial increase in the peak current suggests that the enhancement in conductivity due to the low concentration of AuNPs was relatively limited; conversely, at higher concentrations of AuNPs, the limited number of active sites on the surface of the MGCE and possible aggregation impeded electron transfer. Therefore, 5 mg/mL of αFO/FFO@Au was chosen as the optimal concentration. The optimization of Apt concentration is displayed in Figure 6B; as the Apt concentration increases, the peak current decreases steadily until saturation is achieved at 4 μM of Apt. The initial reduction in peak current indicates increased Apt immobilization on the αFO/FFO@Au MNCs via Au–S bonds; saturation occurs when all available binding sites on the surfaces of the αFO/FFO@Au MNCs are occupied. Beyond this saturation point, further increase in Apt concentration only produced minimal changes in the current response; therefore, the Apt concentration of 4 μM was selected as the optimal value.

The optimizations of the incubation temperature and incubation time for HIF-1α protein are displayed in Figures 6C,D, respectively; both profiles exhibit initial decreases followed by subsequent increases in the peak currents. The initial reductions observed in the peak curves are attributable to the increases in HIF-1α protein and αFO/FFO@Au-Apt/BSA with increases in the incubation temperature and incubation time; here, the peak currents decrease gradually owing to the poor electrical conductivity of HIF-1α protein. However, when the incubation temperature exceeds 37 °C, the higher temperature might reduce the activity of the HIF-1α protein and even prompt its inactivation, leading to reduction of the peak current. Similarly, as the incubation time increases, the quantity of HIF-1α protein captured by the aptamer increases gradually, causing a reduction in the current response (0–45 min). Nevertheless, the lengthy incubation time prompts HIF-1α activity reduction and subsequent inactivation, resulting in an increase in the peak current (45–90 min). Herein, 37 °C and 45 min were employed as the optimal values for incubation of the HIF-1α protein. Although pH plays an important role in aptamer-target recognition, the target matrix of the biosensor is human serum with a fixed physiological pH of 7.35–7.45; therefore, all measurements were performed in PBS at a pH of 7.4 without additional pH gradient optimization.

### 3.7 Analytical performance of the aptamer nanobiosensor

**FIGURE 7:**
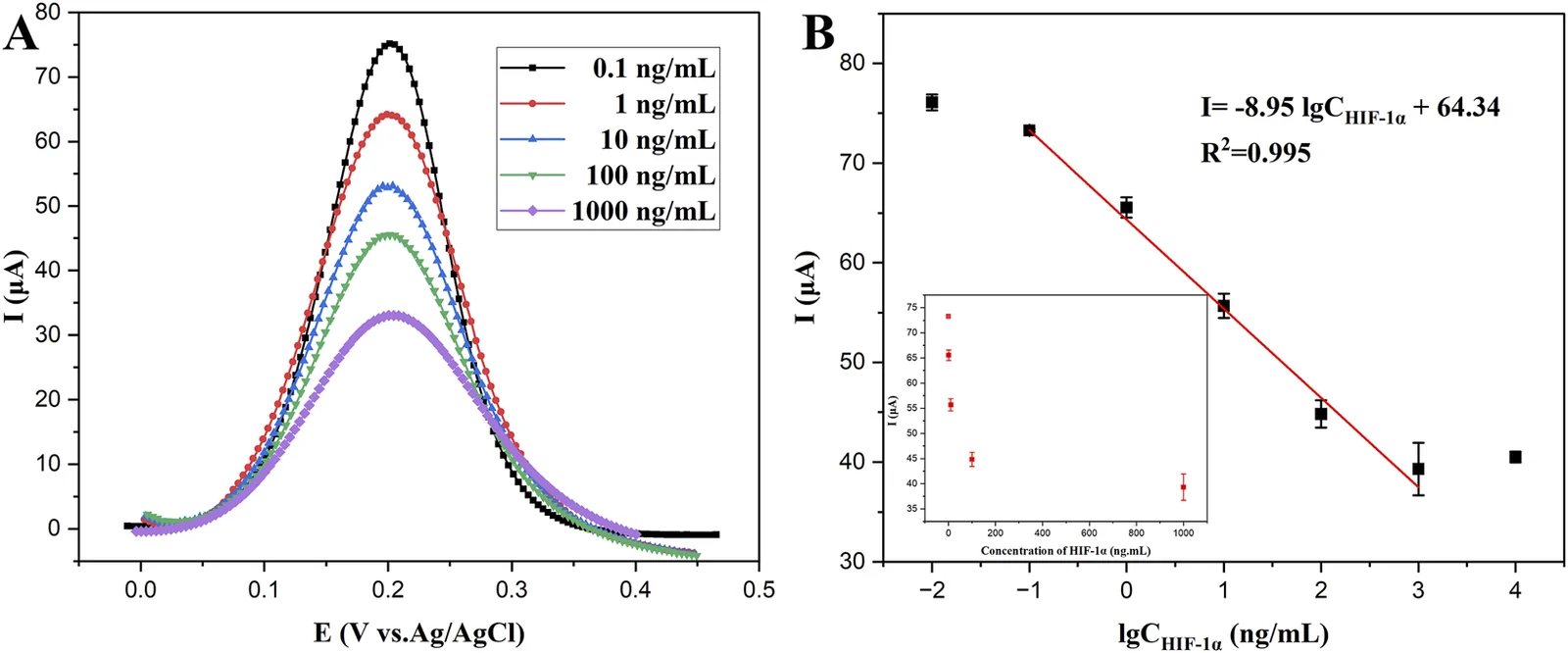
**(A)** Differential pulse voltammetry responses at HIF-1α concentrations of 0.1–1,000 ng/mL and **(B)** their linear fitting; the 0.01 and 10,000 ng/mL concentrations are beyond the linear range for the peak current vs. logarithm of HIF-1α concentration curve, and the inset in **(B)** shows the peak current vs. HIF-1α concentration relationship.

Based on the optimized construction conditions, we further investigated the linearity, anti-false-positive capability, selectivity, stability, reproducibility, and real-sample analytical performance of the aptamer nanobiosensor for detecting HIF-1α protein. The DPV curves for the various HIF-1α concentrations (0.1, 1, 10, 100, and 1,000 ng/mL) are displayed in Figure 7A, which show that the peak current declines with increase in HIF-1α concentration. The correlation between the peak current and logarithm of the HIF-1α concentration is presented in Figure 7B; for the HIF-1α concentration range of 0.1–1,000 ng/mL, the linear regression equation was determined as I = −8.95 (±0.3271) lg C + 64.34 (±0.3335) with fitting variance R^2^ = 0.995, demonstrating excellent linear and negative correlation. The corresponding non-linear relationship is shown in the inset of Figure 7B. The limit of detection (LOD) was calculated to be 0.112 ng/mL, and the limit of quantification (LOQ) was 0.373 ng/mL, based on Equations 5, 6 below:

$$LOD=\frac{3\delta }{k},$$ (5)

$$LOQ=\frac{10\delta }{k},$$ (6)

where δ is the SD of the intercept, and k is the slope of the calibration curve.

Comparing the detection methods for HIF-1α listed in Table 2, the proposed biosensor possesses a relatively wider linear detection range but suffers from a less favorable LOD. Future optimizations could be realized by introducing signal amplification modules in the proposed system to improve the detection sensitivity. Despite the moderate sensitivity, the proposed method exhibits prominent merits, including facile operation, short testing time, and low cost.

**TABLE 2:** Comparison of different methods for HIF-1α detection.

Method	Linear range	Limit of detection	References
SPRi biosensors	5–100 pg/mL	0.09 pg/mL	Zielinska et al. (2024)
Colorimetric determination	0.3–200 pg/mL	0.2 pg/mL	Wang et al. (2019)
Luminescent switch-on detection	0–80 nM	−	Lu et al. (2016)
Electrochemical aptamer nanobiosensor	0.1–1,000 ng/mL	0.112 ng/mL	This work

**FIGURE 8:**
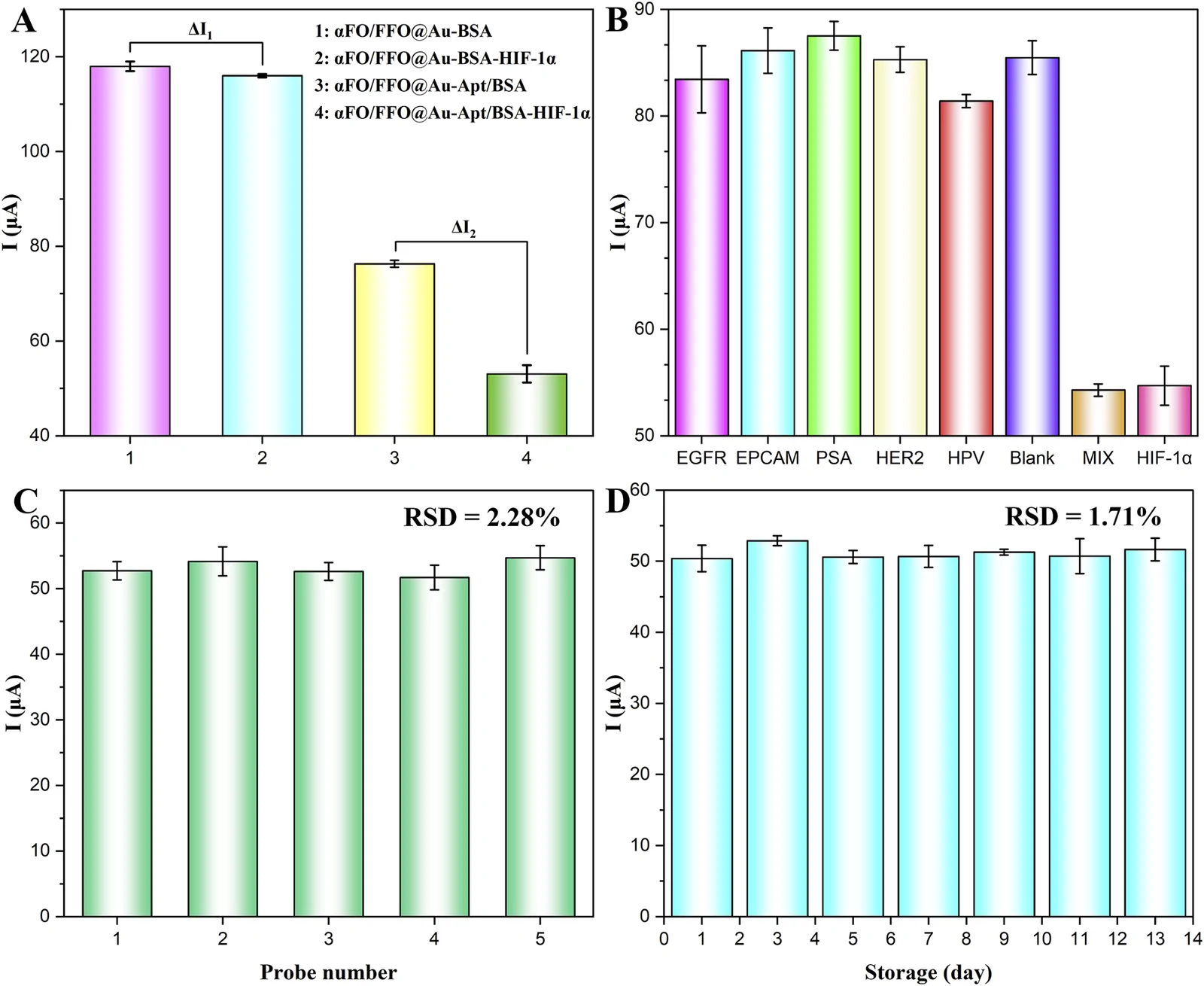
**(A)** False-positive control and **(B)** selectivity experiments of the aptamer nanobiosensor for 10 ng/mL concentrations of HIF-1α, EGFR, EPCAM, HER2, HPV, PSA, and their mixture; **(C)** reproducibility of the aptamer nanobiosensors for five repeated determinations of HIF-1α under optimized conditions, and **(D)** stability of the aptamer nanobiosensor over a period of 13 d.

To ensure detection reliability and detection precision, we investigated the anti-false-positive capability, selectivity, reproducibility, and stability of the aptamer nanobiosensor. The extent of non-specific adsorption was validated by comparing the current changes (ΔI) induced by HIF-1α capture in groups with and without aptamers, which effectively avoids false-positive responses. As shown in Figure 8A, ΔI_1_ is minimal and markedly smaller than ΔI_2_, demonstrating weak non-specific adsorption and limited false-positive interference. Various tumor markers with identical concentrations of 10 ng/mL, including HIF-1α, prostate-specific antigen (PSA), human papillomavirus (HPV), human epidermal growth factor receptor 2 (HER2), epithelial cell adhesion molecule (EPCAM), and epidermal growth factor receptor (EGFR), were measured along with a mixture group and a blank group; these results are shown in Figure 8B and illustrate that the proposed aptamer nanobiosensor exhibits excellent selectivity as it specifically recognizes only the HIF-1α protein. Under the optimized conditions, five replicate determinations of HIF-1α were performed; these results are shown in Figure 8C and indicate that the current responses remain stable with an RSD of 2.28%. Thereafter, seven groups of αFO/FFO@Au-Apt/BSA aptamer nanobiosensors were constructed and incubated with HIF-1α, and these nanobiosensors were evaluated for stability once every 2 d. The current signals were observed to be stable on day 13 with an RSD of 1.71% (Figure 8D), revealing the dependability of the aptamer nanobiosensor for the detection of HIF-1α protein.

Human serum is a complex matrix consisting of inorganic ions, proteins, lipids, glucose, metabolites, hormones, circulating nucleic acids, and hemolytic ingredients. To verify the prospects of the aptamer nanobiosensor for detecting HIF-1α under clinical test conditions, samples spiked with various concentrations of this protein (1, 10, and 100 ng/mL) in human serum solutions prepared by diluting human serum 20-fold with 10 mM PBS (pH = 7.4) were used for detection according to the proposed method; these detection data are enumerated in Table 3. The recoveries for the spiked concentrations in real samples ranged from 97.71% to 107.47%, with the RSDs not exceeding 3.47%; these results demonstrate that the electrochemical aptamer nanobiosensor holds considerable potential for the detection of HIF-1α in real serum samples. In summary, the proposed detection method has many advantages, including operational convenience, short assay time, high accuracy, excellent selectivity, reproducibility, and stability, demonstrating promising application prospects in clinical tests.

**TABLE 3:** Analytical results for the determination of HIF-1α in spiked human serum samples (n = 3).

Added (ng/mL)	Found (ng/mL)	Relative standard deviation (%)	Recovery rate (%)
1	0.98	2.15	97.71
10	10.21	3.47	102.07
100	107.47	1.87	107.47

## 4 Conclusion

1. αFO/FFO MHNRs were successfully prepared through the hydrothermal and calcination-reduction processes. The αFO/FFO MHNRs were calcined at 500 °C for 4 h in the ratio of 16:1 for glucose and β-FeOOH NRs fabricated at a hydrothermal temperature of 220°C for 12 h; these MHNRs had an average length of approximately 135 nm, average diameter of approximately 55 nm, and Ms of 29.8 emu/g.
2. AuNPs of diameter 6 nm were loaded onto the surfaces of the αFO/FFO MHNRs to reinforce the electron transfer capacity and realize secure immobilization of Apt via Au–S bonds; thus, we successfully constructed an electrochemical aptamer nanobiosensor for the detection of HIF-1α protein. The aptamer nanobiosensor revealed a favorable linear relationship over the large range of 0.1–1,000 ng/mL, with R^2^ of 0.995, LOD of 0.112 ng/mL, and LOQ of 0.373 ng/mL. Meanwhile, the aptamer nanobiosensor showed excellent sensitivity, faithful reproducibility with an RSD of 2.28%, reliable 13-d stability with an RSD of 1.71%, and accredited detection in real samples with recoveries of 97.71%–107.47% and RSDs ≤ 3.47%. These analytical performance results suggest the promising prospects of the proposed aptamer nanobiosensor under clinical test conditions.

## Data Availability

The original contributions presented in the study are included in the article/supplementary material; further inquiries can be directed to the corresponding authors.
